# FSH Receptor Asn680Ser Polymorphism Modulates Intrafollicular Nitric Oxide Bioavailability and Ovarian Responsiveness During IVF

**DOI:** 10.3390/ijms27052452

**Published:** 2026-03-06

**Authors:** Charalampos Voros, Diamantis Athanasiou, Despoina Mavrogianni, Ntilay Soyhan, Georgia Panagou, Maria Sakellariou, Georgios Papadimas, Fotios Chatzinikolaou, Eleni Sivylla Bikouvaraki, Georgios Daskalakis, Kalliopi Pappa

**Affiliations:** 1 First Department of Obstetrics and Gynecology, National and Kapodistrian University of Athens, 106 79 Athens, Greece; diamathan16@gmail.com (D.A.); depy.mavrogianni@yahoo.com (D.M.); dr.georgepapadimas@gmail.com (G.P.); fotischatzin@auth.gr (F.C.); kalliopi.pappa20@gmail.com (K.P.); 2 IVF Athens Reproduction Center V.A., 151 23 Maroussi, Greece; dilay000@hotmail.com (N.S.); zetat_@hotmail.com (G.P.); marisakellariou@gmail.com (M.S.); 3 Laboratory of Cell and Gene Therapy, Biomedical Research Foundation of the Academy of Athens Greece (BRFAA), 115 27 Athens, Greece; kalliopi.pappa11@gmail.com

**Keywords:** infertility, Asn680Ser polymorphism, FSH receptor, nitric oxide, IVF

## Abstract

In vitro fertilisation (IVF) has significant hurdles due to individual differences in ovarian response during controlled ovarian stimulation. The Asn680Ser polymorphism of the follicle-stimulating hormone receptor (FSHR) is linked to varying ovarian sensitivity to FSH. However, its relationship with intrafollicular redox signalling remains unclear. Nitric oxide (NO) is a crucial compound that functions inside follicles and participates in angiogenesis, steroidogenesis, and oocyte competence. This prospective observational research classified women undergoing IVF into Asn allele carriers (Asn/Asn and Asn/Ser) and Ser/Ser homozygotes, according to the FSHR Asn680Ser polymorphism. The groups were assessed according to follicular fluid nitric oxide metabolites (NO_2_-NO_3_), fertilisation results, ovarian response indicators, and hormonal profiles. No substantial variation was seen between baseline and trigger-day hormone levels. In contrast, Ser/Ser individuals had a significantly higher total count of recovered oocytes, an elevated number of metaphase II oocytes, and enhanced fertilisation outcomes relative to carriers. The Ser/Ser group demonstrated increased intrafollicular NO_2_-NO_3_ concentrations. This difference was not statistically significant. These results link FSH receptor genetics to functional follicular competence, indicating that the FSHR Asn680Ser polymorphism is associated with differing ovarian responsiveness during IVF and may affect intrafollicular nitric oxide bioavailability.

## 1. Introduction

Controlled ovarian stimulation (COS) is a crucial component of in vitro fertilisation (IVF). The objective is to maximise oocyte retrieval and enhance the likelihood of producing viable embryos by promoting the concurrent development of several follicles [1]. COS aims to overcome the physiological limitation of monofollicular recruitment while preserving oocyte quality and reducing treatment-related risks by pharmacologically modifying the hypothalamic-pituitary-ovarian axis [2]. Despite significant advancements in stimulation techniques and individualised dosage adjustments based on age, ovarian reserve indicators, and body mass index, considerable variability persists in the ovarian response of IVF patients. This variability is often seen in individuals with similar stimulation protocols and baseline hormonal levels [3].

Conventional clinical or hormonal metrics alone cannot adequately account for the interindividual differences in follicular recruitment, oocyte maturation, and fertilisation results [4]. An expanding corpus of research indicates that genetic variations and other variables inside the ovaries significantly influence follicular sensitivity to gonadotropin stimulation. The effectiveness of ovarian follicles in reacting to exogenous FSH may be affected by genetic differences linked to gonadotropin signalling, steroidogenesis, angiogenesis, and intracellular metabolic pathways [5]. The growing recognition of genetic factors as crucial influences on ovarian responsiveness variability during COS highlights the need to incorporate molecular and genetic components into modern personalised IVF treatment models.

FSH is crucial for regulating ovarian function as it interacts with the follicle-stimulating hormone receptor (FSHR), a G protein-coupled receptor mostly located on granulosa cells inside the ovarian follicles [6]. Upon FSHR binding to FSH, protein kinase A (PKA) is activated, initiating intracellular signalling pathways mostly involving the synthesis of cyclic adenosine monophosphate (cAMP). This sequence of events initiates many downstream transcriptional and post-transcriptional mechanisms that regulate critical ovarian activities, including granulosa cell proliferation, steroidogenesis, and follicular maturation. These mechanisms are essential for fertilisation, oocyte maturation, and the first phases of embryonic development [7].

Genetic and extrinsic variables, including hormonal environment, affect FSHR functioning and, subsequently, the ovarian response to FSH during COS. Researchers have mostly examined the Asn680Ser (rs6166) variation in the FSHR gene [8]. The Ser680 allele is associated with a modification in the receptor’s structure and function, resulting in an amino acid substitution at position 680 [9]. Homozygosity for the Ser680 allele modifies FSHR sensitivity, resulting in alterations in FSH receptor binding affinity, receptor activation, and downstream signalling. These modifications may affect ovarian responsiveness to gonadotropins, possibly influencing the clinical results of IVF and requiring adjustments in gonadotropin dose for optimum ovarian response [10]. Research suggests that the Ser680 allele may affect ovarian responsiveness to stimulation, perhaps clarifying the differences in ovarian function and IVF success across people. Thus, the FSHR Asn680Ser polymorphism is a significant genetic factor influencing individual ovarian responses and may act as a predictor for customised IVF treatment methods [11]. The intrafollicular microenvironment, integrating systemic hormonal signals with local vascular, metabolic, and redox signals, is crucial for optimal follicular development alongside traditional endocrine control. This meticulously regulated milieu governs granulosa cell-oocyte communication, follicular perfusion, oxygen supply, and nutrient accessibility. This is essential for the maturation of the egg in both the nucleus and the cytoplasm. Despite sufficient circulating gonadotropin levels, the disruption of local signalling networks might hinder folliculogenesis, highlighting the significance of intrafollicular regulating systems [12].

In this context, nitric oxide (NO) has emerged as a significant signalling chemical inside follicles, exerting various effects on organisms. Nitric oxide synthase isoforms present in granulosa cells, theca cells, and follicular endothelium produce nitric oxide [13]. NO regulates angiogenesis, vascular tone, and follicular blood flow, influencing the supply of oxygen and nutrients to the growing follicle. NO has been associated with the regulation of meiotic development, mitochondrial activity, and cytoskeletal integrity in the oocyte [14]. It directly facilitates granulosa cell steroidogenesis via cyclic guanosine monophosphate (cGMP)-dependent mechanisms. Owing to its short biological half-life, the stable oxidative metabolites nitrate and nitrite (NO_2_-NO_3_) are often used to evaluate NO bioavailability in the follicular compartment [15]. These metabolites provide a dependable real-time perspective on redox and vascular dynamics inside the follicle. The role of NO in follicular competence during controlled ovarian stimulation is supported by growing clinical and experimental evidence that shows elevated intrafollicular NO_2_-NO_3_ concentrations are associated with improved ovarian response, enhanced oocyte maturity, higher fertilisation rates, and increased steroidogenic activity [13]. However, the processes governing intrafollicular nitric oxide generation and bioavailability remain little understood. A notable gap in understanding the molecular control of ovarian responsiveness during IVF is the lack of comprehensive studies examining the influence of genetic variability in FSH receptor signalling on NO-mediated intrafollicular pathways [16].

The signalling of FSHR is essential for the regulation of granulosa cell proliferation, steroidogenic function, and follicular maturation. Moreover, NO is recognised for its impact on the intrafollicular vascular and redox milieu [17]. Consequently, it is physiologically feasible that genetic diversity in FSHR function may influence intrafollicular NO dynamics. Modifications in FSHR-mediated intracellular signalling may affect follicular perfusion, steroidogenesis, and oocyte developmental competence by altering downstream pathways linked to endothelial nitric oxide synthase activation, cyclic nucleotide signalling, and redox homeostasis within the follicular compartment [18]. Despite similar hormonal stimulation regimens, clinically observed differences in ovarian responsiveness and oocyte quality among women receiving controlled ovarian stimulation may be explained by gene-microenvironment interactions [19]. Thus, clarifying the association between FSHR polymorphisms and intrafollicular NO bioavailability may improve comprehension of ovarian responsiveness and provide mechanistic insights into the molecular foundations of interindividual heterogeneity in COS outcomes. The discovery of genetic factors affecting functional follicular indicators may enable the creation of sophisticated, personalised stimulation techniques for assisted reproduction [20]. Our study aimed to examine the relationship between the FSHR Asn680Ser polymorphism and intrafollicular nitric oxide bioavailability, measured by follicular fluid NO_2_-NO_3_ levels, as well as its correlation with ovarian response metrics and embryological outcomes in women undergoing in vitro fertilisation.

## 2. Results

The conclusive study included women who had controlled ovarian stimulation and oocyte extraction, together with comprehensive hormonal, embryological, follicular fluid, and FSHR genotyping data. The subjects were categorised into two groups according to the FSHR Asn680Ser polymorphism: homozygous Ser/Ser individuals (*n* = 23) and Asn allele carriers (Asn/Asn *n* = 40 and Asn/Ser *n* = 19). We analysed hormonal profiles, ovarian response parameters, embryological outcomes, and nitric oxide metabolite concentrations inside follicles across several genotype groups. Baseline endocrine and demographic variables were originally evaluated to guarantee comparability across groups. Subsequently, the FSHR genotype was examined in connection to ovarian response indicators, such as follicular development, oocyte yield, and oocyte maturity. The number of 2PN embryos obtained after insemination was used to assess fertilisation results. The amounts of NO_2_-NO_3_ in follicular fluid were compared among genotype groups to assess the bioavailability of nitric oxide inside the follicle. The Statistical Analysis section delineates the methodology used for statistical comparisons using either parametric or non-parametric tests. The findings are shown as mean ± standard deviation. The baseline demographic, hormonal, and embryological characteristics of the study population are summarised in Table 1.

Table 1 presents the baseline hormonal, embryological, and demographic characteristics of the whole research cohort. The conclusive investigation included 89 women undergoing controlled ovarian stimulation for in ΙVF. The cohort exhibited an average age of 38.4 ± 6.0 years and had hormonal and embryological traits typical of a standard IVF population. The average concentrations of NO_2_-NO_3_ in follicular fluid were within normal limits, despite the average quantities of retrieved oocytes, MII oocytes, and 2PN embryos indicating a favourable ovarian response and potential for fertilisation. These foundational characteristics provide a representative framework for future genotype-based comparisons and improve the internal validity of the research population. To determine whether differences in ovarian response and embryological outcomes are associated with systemic endocrine fluctuations, baseline and trigger-day hormonal parameters were analysed among women classified by the FSHR Asn680Ser polymorphism. Table 2 contrasts the blood concentrations of progesterone, luteinizing hormone, oestradiol, and follicle-stimulating hormone between individuals possessing the Asn allele and those homozygous for the Ser allele.

We examined the baseline and trigger-day hormonal parameters (Table 2) of women who were homozygous for the Ser/Ser version of the FSHR Asn680Ser polymorphism and those who had the Asn allele (Asn/Asn and Asn/Ser). No statistically significant changes were seen in blood levels of FSH, E2, LH, and PRG across genotype groups (all *p* > 0.05). The findings indicate similar systemic endocrine profiles between the two groups at baseline and during ovulation induction, implying that variations in circulating gonadotropin or steroid hormone levels are unlikely to contribute to subsequent differences in ovarian response and embryological outcomes. Ovarian response parameters and embryological outcomes were subsequently compared between FSHR Asn allele carriers and Ser/Ser homozygous women, as presented in Table 3.

**Figure 1:**
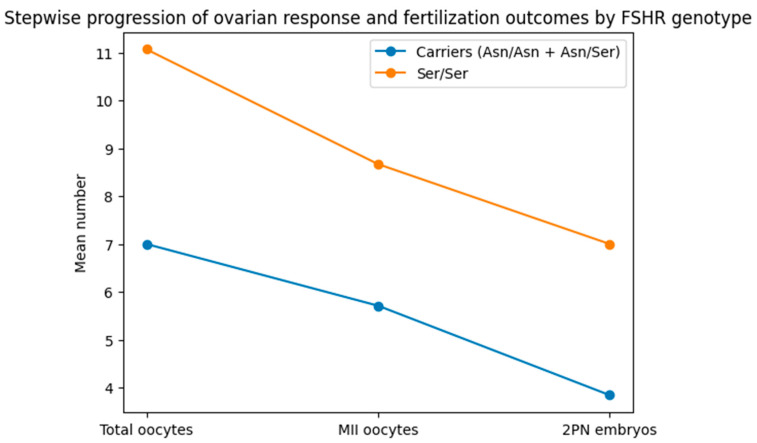
Women possessing the FSHR Asn allele and those homozygous for Ser/Ser exhibited average values for total oocytes recovered, MII oocytes, and 2PN embryos. Ser/Ser individuals consistently demonstrated higher mean values than carriers at every stage, indicating improved fertilisation effectiveness and ovarian responsiveness.

Table 3 presents a comparison of ovarian response and embryological outcomes based on the FSHR Asn680Ser genotype. Women possessing the Asn allele and those homozygous for Ser/Ser exhibited comparable durations for follicular development and ovulation induction (18.92 ± 2.79 mm vs. 18.27 ± 1.44 mm, *p* = 0.593). In contrast, quantitative ovarian response characteristics showed considerable variability. The total oocyte count was much greater in Ser/Ser people than in carriers (11.07 ± 7.12 vs. 7.00 ± 4.15, *p* = 0.030), suggesting a more vigorous ovarian response to controlled stimulation. A same pattern was seen in oocyte maturity, with Ser/Ser women exhibiting a substantially greater quantity of MII oocytes than carriers (8.67 ± 4.95 vs. 5.71 ± 3.22, *p* = 0.046). The fertilisation results were markedly better in the Ser/Ser group, corresponding with the improved oocyte quantity and maturity. Women homozygous for Ser/Ser exhibited almost double the number of properly fertilised 2PN embryos compared to carriers (7.00 ± 4.95 vs. 3.84 ± 2.62, *p* = 0.016). The findings indicate statistically significant genotype-dependent differences in early embryological performance and ovarian responsiveness, independent of changes in follicular size. Figure 1 depicts a sequential comparison of the several phases of oocyte development, highlighting the genotype-dependent changes in ovarian response and fertilisation results.

By analysing the concentrations of NO_2_-NO_3_ in follicular fluid across several FSHR genotype groups, we gained enhanced insight into the availability of nitric oxide inside the follicles (Table 4).

Analysis of nitric oxide metabolites in follicular fluid revealed that women homozygous for the Ser/Ser genotype had elevated mean NO_2_-NO_3_ concentrations compared to those possessing the Asn allele (123.57 ± 121.58 vs. 85.16 ± 76.99 µmol/L). However, this difference was not statistically significant (*p* = 0.319). In both genotype groups, the distribution of data showed significant interindividual variability, with Ser/Ser individuals showing a wider range and higher mean concentrations of intrafollicular nitric oxide metabolites.

## 3. Discussion

Our research reveals that the FSHR Asn680Ser polymorphism is linked to significant differences in ovarian responsiveness and early embryological outcomes under controlled ovarian stimulation for in vitro fertilisation. Women homozygous for the Ser/Ser variation had a much superior ovarian response relative to Asn allele carriers, as shown by an elevated total oocyte output, a larger quantity of mature metaphase II oocytes, and an increased prevalence of properly fertilised two-pronuclear embryos [11,21]. Despite the follicular diameters and hormonal profiles on baseline and trigger days being comparable between genotype groups, these discrepancies were noted, suggesting that systemic endocrine variation or changes in follicular development do not explain the observed effects [22]. Concurrently, Ser/Ser individuals exhibited numerically higher intrafollicular nitric oxide metabolite concentrations, but this difference did not reach statistical significance. This variation was not statistically significant [23]. Collectively, these findings indicate that follicular function and oocyte competence, rather than variations in circulating hormone levels, may be the focal point of FSHR genotype-dependent regulation of ovarian response. The continual enhancement of results across the various phases of oocyte maturation and fertilisation highlights a possible influence of FSHR genetic variation on the functional intrafollicular microenvironment during IVF. FSHR polymorphism may influence ovarian function through different molecular mechanisms unrelated to nitric oxide-mediated signalling, given the lack of substantial differences in nitric oxide metabolite levels among genotype groups.

### 3.1. FSHR Asn680Ser Polymorphism and Ovarian Responsiveness During Controlled Ovarian Stimulation

The Ser680 allele is linked to altered ovarian responsiveness to exogenous follicle-stimulating hormone, commonly indicated by elevated basal FSH levels, enhanced gonadotropin utilisation, and, in multiple cohorts, a diminished number of retrieved oocytes, as documented by de Castro et al., Jun et al., Yao et al., and Huang et al. [21,24,25]. These data corroborate the concept that the Ser680 mutation modifies receptor effectiveness rather than indicating diminished ovarian reserve. Tang et al., Pabalan et al., and Prodromidou et al. demonstrate significant variability across demographic groups, ethnic backgrounds, and COS methods via extensive meta-analyses and systematic reviews [8,26,27]. They also demonstrate that stimulation settings vary according to genotype. The biological impact of the Asn680Ser polymorphism is neither uniform nor conclusive, as this variability highlights.

Furthermore, Jun et al., Huang et al. demonstrate that Ser/Ser carriers often need increased FSH doses and have reduced oestradiol responses during the trigger phase, indicating altered steroidogenic sensitivity of granulosa cells [10,25]. Yao et al. and Tang et al. demonstrate a disparity between hormonal function in the body and the efficacy of the reproductive system [8,21]. These hormonal changes do not always result in diminished rates of fertilisation or pregnancy. Baldini et al. provide clinically significant evidence that Ser680 carriage may elicit an atypical ovarian response in women with normal anti-Müllerian hormone levels and antral follicle counts, demonstrating that FSHR polymorphisms operate independently of conventional ovarian reserve indicators [28].

Alviggi et al., Conforti et al., and Humaidan et al. elucidate that ovarian response is a complex phenotype influenced by hormonal, functional, and genetic factors [23,29,30]. While advising against their exclusive clinical use, multicentre and consensus-derived results support the integration of FSHR polymorphisms into a comprehensive pharmacogenomic framework. Genotype-guided stimulation studies, exemplified by Hjelmér et al., validate the functional importance of rs6166 in certain clinical scenarios by demonstrating that altering gonadotropin type based on FSHR genotype may improve cumulative pregnancy and live birth rates [31].

Despite the follicular diameters being identical at the time of ovulation induction, our findings indicate that women homozygous for the Ser680 allele had much more retrieved oocytes, metaphase II oocytes, and two-pronuclear embryos than anticipated [11]. This pattern indicates that the ovaries are not less sensitive; rather, it demonstrates that follicular recruitment is sustained and intrafollicular competence is enhanced [32]. The lack of genotype-dependent differences in follicular size indicates that the Ser/Ser impact in our cohort primarily affects oocyte maturation and fertilisation efficiency, rather than follicular development dynamics. These data challenge the simplistic categorisation of the Ser680 allele as a “poor-response” genotype, suggesting a more complex, context-dependent function [33].

By illustrating that follicle-stimulating hormone receptor signalling transcends the traditional cAMP-protein kinase pathway A central line around which an object rotates. Casarini et al., Hunzicker-Dunn et al., and Gloaguen et al. provide a mechanical elucidation for this divergence [34,35,36]. The simultaneous activation of PI3K/AKT, ERK1/2, p38 MAPK, and β-arrestin-dependent pathways governs granulosa cell viability, metabolic adaptability, steroidogenesis, and oocyte-supportive activities. Landomiel et al. and Ulloa-Aguirre et al. demonstrate that FSHR variants can affect signalling bias, intracellular compartmentalisation, and receptor trafficking, thus altering the qualitative response to FSH stimulation without necessarily impacting ligand affinity or cell surface receptor expression [37,38].

It is biologically plausible that the Ser680 receptor variation may selectively activate non-canonical pathways that promote oocyte maturation and fertilisation competence under certain stimulation circumstances within this signalling framework [39]. Despite other studies reporting diminished endocrine sensitivity, this explanation aligns with our discovery that Ser/Ser carriers exhibited superior MII and 2PN results [40]. This signalling bias may explain why follicular growth metrics and systemic endocrine indicators fail to effectively reflect genotype-specific changes in intrafollicular functioning [41].

### 3.2. FSHR Asn680Ser Polymorphism, Intrafollicular Nitric Oxide Bioavailability, and Oocyte Competence

Sugino et al., Manau et al., and Fıçıcıoğlu et al. were the pioneers in comprehensively delineating nitric oxide metabolites in human follicular fluid during controlled ovarian stimulation [42,43,44]. They demonstrated that NO_2_-NO_3_ concentrations reflect local follicular physiology instead of systemic hormonal patterns. Initial pessimism about the therapeutic efficacy of nitric oxide arose from these studies’ inability to reliably predict pregnancy or the overall ovarian response. However, the lack of segmentation according to intrafollicular signalling heterogeneity or genetic background in these research may have concealed physiologically meaningful associations. These preliminary experiments demonstrated that nitric oxide is not only a passive oxidative byproduct but an active paracrine regulator in the follicular milieu [13].

Barrionuevo et al. and Vignini et al. provided a more sophisticated analysis, revealing that increased follicular NO_2_-NO_3_ levels were associated with lower fertilisation rates, decreased embryo cleavage, and suboptimal embryo shape [45,46]. The data suggest that nitrosative stress, mitochondrial malfunction, or alteration of spindle dynamics may negatively impact oocyte competence owing to excessive nitric oxide generation [47].

Staicu et al. significantly advanced the science by delineating the distinction between qualitative oocyte competency and quantitative ovarian response [20]. The levels of nitrite and nitrate in oocyte donors correlated with the proportion of metaphase II oocytes and the likelihood of embryo implantation, but not with the overall oocyte yield. This discovery directly challenges the characterisation of nitric oxide as a binary “good” or “bad” indicator, instead advocating for a paradigm in which nitric oxide regulates developmental potential and meiotic competence [16]. The thorough analyses by Budani and Tiboni et al. incorporate both animal and human data, illustrating that nitric oxide regulates angiogenesis, follicular perfusion, mitochondrial respiration, steroidogenesis, and meiotic progression in a concentration and timing dependent manner, strongly supporting this interpretation [48].

The upstream factors influencing the intrafollicular availability of nitric oxide during IVF cycles are little understood, despite growing evidence of its functional importance. Specifically, gonadotropin receptor signalling has received less attention in human research. Casarini et al., Hunzicker-Dunn et al., and Gloaguen et al. provide a compelling molecular explanation for the association between FSH receptor activation and nitric oxide regulation [34,35,36]. These results show that FSHR signalling is markedly pleiotropic, including not only the classical cAMP-PKA route but also PI3K/AKT, ERK1/2, p38 MAPK, and calcium-dependent pathways, all of which are acknowledged regulators of endothelial nitric oxide synthase production and activity.

Chen et al. and Hunzicker-Dunn et al. demonstrate that FSH-mediated stimulation of the PI3K/AKT pathway results in the direct phosphorylation of eNOS at activating sites, so demonstrating a direct molecular link between nitric oxide synthesis and FSH signalling [12,35]. Landomiel et al. and Ulloa-Aguirre et al. demonstrate that various FSHR types may alter signalling bias, β-arrestin recruitment, and receptor mobility, hence affecting the dynamics of signalling temporally and spatially [37,38]. These results are noteworthy since they suggest that FSHR mutations may indirectly impact nitric oxide bioavailability by modifying the architecture of intracellular signalling rather than influencing ligand binding affinity.

Within this mechanistic framework, our findings present novel human data suggesting a tendency for genotype-dependent variation in intrafollicular nitric oxide levels, despite lacking statistical significance. The mean concentrations of NO_2_-NO_3_ were numerically higher in Ser/Ser carriers than in Asn allele carriers. This difference did not reach statistical significance. The effect’s directionality is physiologically coherent and is magnified when examined in conjunction with embryological outcomes, albeit the difference did not reach statistical significance. This trend is consistent with the results of our prior studies. In the Voros et al. (CART research), Ser/Ser carriers exhibited significantly elevated levels of intrafollicular CART expression [49]. CART is a peptide associated in granulosa cell signalling, angiogenesis, and metabolic control. This indicates that the Ser/Ser genotype may be linked to a more extensive reorganisation of the intrafollicular signalling environment, rather than isolated alterations in a single pathway.

The research conducted by Voros et al. on bariatric surgery and eNOS corroborated this notion by demonstrating that eNOS expression and vascular signalling are contingent upon genotype [14]. Ser/Ser carriers exhibited significant diversity and responsiveness in pathways associated with nitric oxide. These data demonstrate a similar pattern in which intrafollicular mediators, including as CART and nitric oxide, involved in metabolic, vascular, and redox control, are affected by the FSHR Ser/Ser genotype. The lack of a statistically significant difference in NO_2_-NO_3_ concentrations in the present investigation does not negate biological relevance. It underscores the considerable inter-individual heterogeneity in nitric oxide signalling and the possible threshold dependency of its effects. Despite comparable follicular dimensions, Ser/Ser carriers in our cohort exhibited a significantly greater quantity of metaphase II oocytes and two-pronuclear embryos. This indicates that their intrafollicular competence enhanced rather than their follicular development altered. This trend aligns with the findings of Staicu et al. and Barrionuevo et al., who established that nitric oxide had the most pronounced effect on qualitative reproductive outcomes [20,45].

These results explain the contradictory literature linking elevated follicular nitric oxide to both neutral and adverse consequences. Nitric oxide seems to operate within a limited physiological range rather than as a linear indicator. It facilitates meiotic development, angiogenesis, oxygen transport, and mitochondrial efficiency at optimal levels [50]. Excessive nitric oxide may induce nitrosative stress, impairing the functionality of oocytes thereafter. Modifying receptor signalling may facilitate the regulation of nitric oxide synthesis within the optimal range in genetically specified contexts, such as in Ser/Ser FSHR carriers. This would enhance oocyte maturation and fertilisation efficiency without inducing oxidative damage.

### 3.3. Clinical Implications and Translational Perspectives

The significant interindividual variability noted during controlled ovarian stimulation cannot be adequately explained by traditional clinical and endocrine metrics, including chronological age, basal follicle-stimulating hormone levels, anti-Müllerian hormone concentrations, or antral follicle count, as highlighted by Alviggi et al., Conforti et al., and Humaidan et al. [23,29,30]. These parameters remain significant for forecasting future outcomes; however, they primarily assess ovarian reserve rather than ovarian functionality. Their study promotes a paradigm change towards multidimensional patient profile that integrates hormonal, functional, and genetic variables to more precisely reflect the biological variability of ovarian response. In this setting, FSHR polymorphisms, notably the Asn680Ser variation, have consistently altered gonadotropin sensitivity and stimulation dynamics. These authors highlight the need of integrative models that merge genetic background with subsequent functional outcomes, while simultaneously stressing the limited clinical significance of individual polymorphisms.

Tang et al., Pabalan et al., and Prodromidou et al. illustrate through extensive meta-analyses and systematic reviews that the FSHR Asn680Ser genotype is consistently correlated with intermediate stimulation outcomes, encompassing basal FSH levels, total gonadotropin requirements, and, in specific populations, the number of retrieved oocytes [8,26,27]. These studies indicate that genotype is not a reliable predictor of pregnancy or live birth rates. This discrepancy reveals a significant deficiency in existing genotype-based methodologies: they depend on clinical endpoints that are not consistently reliable markers of follicular competency or oocyte developmental potential. Meta-analyses demonstrate significant diversity in research design, ethnicity, and stimulation methods, indicating that the biological effect of FSHR polymorphism is context-dependent and presumably shaped by intrafollicular signalling settings, rather than serving as a uniform predictor.

Hjelmér et al. provide a persuasive illustration of clinical translation by demonstrating that genotype-guided selection of gonadotropin type recombinant vs. urinary FSH leads to markedly improved cumulative pregnancy and live birth rates [31]. Their results suggest that the FSHR Asn680Ser polymorphism functions as a clinically relevant factor in treatment decision-making, rather than just a passive genetic marker. Significantly, therapeutic effect was not visible when genotyping was used alone. It became apparent only when combined with personalised pharmaceutical approaches. This discovery highlights an essential translational principle: genetic information gains clinical relevance only when linked to alterable cellular pathways and therapeutic modifications.

Our results augment this translational paradigm by integrating intrafollicular nitric oxide bioavailability as a functional mediator between FSHR genotype and oocyte competency. Intrafollicular NO_2_-NO_3_ concentrations, in contrast to systemic endocrine indicators, indicate localised vascular, metabolic, and redox dynamics that directly affect granulosa cell function, meiotic development, and fertilisation potential [13,51]. FSHR polymorphism may have clinically important implications at the microenvironmental level, as shown by the finding that Ser/Ser carriers had improved embryological outcomes and modified nitric oxide profiles. The assessment of ovarian response by follicle counts, oestradiol levels, or gonadotropin dosage reveals little impact, underscoring a significant inadequacy in current clinical evaluation techniques [52].

This insight has direct therapeutic ramifications as it challenges the common practice of categorising patients only based on oocyte quantity into “poor,” “normal,” or “hyper” responders. Although advantageous, these categories may oversimplify intricate biological processes and hide genotype-specific differences in oocyte quality. Our findings support the premise that qualitative competency should be assessed, especially within genetically defined subgroups. This concept corresponds with the findings of Staicu et al., Barrionuevo et al., and Vignini et al., which consistently indicate that nitric oxide levels have a more substantial impact on oocyte maturity, fertilisation success, and embryo quality than total oocyte yield [20,45,46]. Utilising intrafollicular biomarkers may enhance physicians’ ability to categorise patients more effectively than traditional ovarian response classifications. It may also assist physicians in making judgements based on developmental competence rather than only the quantity of eggs.

The combination of intrafollicular signalling assessments with FSHR genotyping offers a logical and physiologically sound method for tailored stimulation regimens from a pharmacogenomic standpoint. Altering the type, dosage, or stimulation kinetics of gonadotropins may selectively activate signalling pathways in Ser/Ser carriers that promote vascular health, oocyte maturation, and the proper synthesis of nitric oxide. Conversely, different stimulation dynamics may be necessary to attain similar intrafollicular conditions in people with the Asn allele. These factors align perfectly with the mechanistic findings of Casarini et al., Landomiel et al., and Ulloa-Aguirre et al., which illustrate that FSHR variants affect intracellular pathway activation, signalling bias, and receptor trafficking, all of which dictate the qualitative results of gonadotropin stimulation [34,37,38].

Our prior research further substantiates this integrated methodology. The Ser/Ser genotype was correlated with significantly increased intrafollicular CART expression in the Voros et al. (CART research), so connecting FSHR polymorphism to vascular and metabolic regulators inside the follicular niche [49]. The research conducted by Voros et al. on bariatric surgery and eNOS demonstrated genotype-dependent differences in eNOS expression, highlighting the interplay between genetic background, vascular signalling, and reproductive hormone control [16]. The results, in conjunction with the present findings, support the idea that the FSHR Asn680Ser polymorphism characterises a broader intrafollicular signalling phenotype that includes metabolic, vascular, and redox pathways, rather than only unique endocrine differences.

Previous research has mostly focused on the relationship between the FSHR Asn680Ser polymorphism and clinical ovarian response indicators; this work provides further insights by examining genotype-related differences within the follicular milieu. The measurement of CART expression and nitric oxide metabolites in follicular fluid offers a molecular perspective on the possible impact of FSHR genetic variation on local regulatory processes. This technique elucidates the impact of various genotypes on ovarian function by identifying potential pathways within the follicle that alter its operation.

Our study offers innovative insights into genotype-associated molecular variations within the human follicular microenvironment. Nevertheless, further experimental models and mechanistic investigations are required to elucidate the causal relationships between FSHR polymorphism and intrafollicular regulatory mechanisms. Such studies may clarify the molecular underpinnings of genotype-dependent variability in ovarian function.

## 4. Materials and Methods

This prospective observational research was executed at the IVF Athens Reproduction Centre V.A., Maroussi, Athens, Greece, after clearance from the Scientific and Ethics Committee under Protocol No. EVD 0702/2022 (18 April 2022). The study was conducted in compliance with the principles of the Declaration of Helsinki and all relevant institutional and governmental regulations on human research. Prior to enrolment, each participant provided their written permission. We adhered to the General Data Protection Regulation (GDPR) and institutional privacy protocols when processing and anonymising all personal data.

The research group included women diagnosed with infertility who received COS during IVF therapy at the IVF Athens Reproduction Centre from October 2022 to October 2024. Stringent inclusion and exclusion criteria were established to eliminate confounding factors and guarantee homogeneity within the research sample. Prior to initiating ovarian stimulation, each participant submitted a detailed medical and reproductive history and received a baseline gynaecological examination, including transvaginal ultrasonography, hormonal profile, and an assessment of uterine and ovarian architecture.

Women between the ages of 20 and 42 with functional ovaries, a body mass index (BMI) of 18 to 35 kg/m^2^, and a normal uterine cavity as determined by transvaginal ultrasonography or hysteroscopy were deemed suitable for participation. Normal ovarian reserve characteristics include anti-Müllerian hormone (AMH) levels above 1.1 ng/mL, an antral follicle count (AFC) of no less than 5 follicles per ovary, and regular menstrual cycles ranging between 25 and 35 days. Women undergoing their first or second IVF cycle were eligible, provided their prior cycles did not fulfil the Bologna criterion for inadequate ovarian response. To mitigate potential alterations in follicular physiology resulting from repeated gonadotropin exposure, only women demonstrating a favourable ovarian response in a prior cycle were deemed suitable.

Reasons for ineligibility included severe endometriosis, prior ovarian surgery, chemotherapy or radiation treatment, and a diminished ovarian reserve (AMH < 1.1 ng/mL and AFC < 5). Factors that excluded individuals were uncontrolled diabetes mellitus, thyroid disorders (TSH > 4.5 µIU/mL), autoimmune illnesses (such as antiphospholipid syndrome, Hashimoto’s thyroiditis, and systemic lupus erythematosus), and hypertension requiring pharmacological intervention. Lifestyle variables prohibited included the use of illicit substances, excessive alcohol consumption (exceeding 10 units per week), and smoking (a minimum of 5 cigarettes daily), all of which are recognised as detrimental to fertility. Initially, blood levels of FSH, luteinizing hormone (LH), oestradiol (E2), progesterone (P4), and prolactin (PRL) were assessed. We used thyroid-stimulating hormone (TSH) and free thyroxine (FT4) to assess thyroid function. We assessed metabolic function by measuring fasting glucose and insulin levels. Transvaginal ultrasonography was conducted in the early follicular phase to evaluate ovarian morphology, antral follicle count (AFC), and any uterine or adnexal anomalies. Subsequently, suitable individuals were enrolled and provided with customised COS procedures.

All individuals received COS with a gonadotropin-releasing hormone (GnRH) antagonist technique, chosen for its safety and decreased risk of ovarian hyperstimulation syndrome (OHSS). In formulating stimulation regimens, the patient’s age, BMI, ovarian reserve indicators, and their prior response to stimulation were all considered. Stimulation commenced on cycle days two or three once the ovaries were quiescent. Individuals received subcutaneous injections daily of highly purified human menopausal gonadotropin (HP-hMG; Menopur), recombinant follicle-stimulating hormone (rFSH; Gonal-F or Puregon), or a combination of both. Initial dosages of gonadotropins, ranging from 150 to 300 IU, were dynamically adjusted using step-up or step-down procedures based on blood oestradiol levels and follicular growth.

To induce the ultimate maturation of oocytes, either recombinant human chorionic gonadotropin (r-hCG, 250 µg) or a GnRH agonist (triptorelin, 0.2 mg) was administered, contingent upon the risk level for OHSS. Oocyte retrieval was conducted 36 h post-trigger with little sedation using transvaginal ultrasound-guided aspiration.

Luteal phase support, clinical pregnancy definition, fertilisation evaluation, embryo grading, embryo transfer procedures, and embryo culture were executed in accordance with recognised guidelines. Conventional techniques were used to obtain peripheral venous blood samples and follicular fluid (FF). FF was collected during oocyte retrieval while implementing measures to prevent blood contamination. Only transparent, non-hemorrhagic follicular fluid samples from follicles that produced a mature metaphase II (MII) oocyte were included. To reduce inter-follicular variability, a single representative follicular fluid sample was chosen for each patient, ideally from the biggest developed follicle. The samples were placed in tubes devoid of RNase or DNase, centrifuged at 3000× *g* for 10 min at 4 °C, and thereafter kept at −80 °C until analysis was conducted.

The Griess reaction was used to quantify the concentrations of nitrate (NO_3_^−^) and nitrite (NO_2_^−^) in follicular fluid, therefore indirectly indicating the availability of nitric oxide. We used a commercial Griess Reagent System (Promega, Catalogue #G2930) to quantify the total nitric oxide metabolites (NOx = NO_3_^−^ + NO_2_^−^). Calibration curves were constructed with standards of sodium nitrate and sodium nitrite. Each sample was examined twice, and the coefficients of variation were below 10% both intra-assay and inter-assay. Starting on the fifth day of stimulation, serial transvaginal ultrasonography was used to assess endometrial characteristics and follicular maturation. Prior to the onset of ovulation, the endometrial thickness was assessed in the sagittal plane, while the dimensions, growth rate, and timing of the follicles were documented.

Peripheral venous blood sample were obtained from each participant for genetic investigation. We used conventional techniques to extract genomic DNA from whole blood. Validated molecular approaches were used to genotype the FSHR Asn680Ser (rs6166) polymorphism. Participants were categorised into two groups according to their FSHR genotype: homozygous Ser/Ser or carriers of the Asn allele (Asn/Asn and Asn/Ser). The genotype distribution was assessed for consistency to enable later comparison analysis. The main outcome measures were fertilisation outcomes (two-pronuclear embryos) and ovarian response characteristics (total oocytes recovered, number of metaphase II oocytes). Secondary exploratory studies were conducted to examine intrafollicular NO_2_-NO_3_ levels and their correlation with ovarian and embryological variables.

The statistical analysis was conducted using IBM SPSS Statistics version 31. We used the Kolmogorov–Smirnov test to determine normality. Continuous variables were given as median (interquartile range) or mean ± standard deviation where applicable. We used either the Mann–Whitney U test or the independent-samples t-test to compare the genotype groups. The Pearson and Spearman coefficients were used to assess correlations. All tests were two-tailed, with a significance threshold set at *p* < 0.05. Due to several individuals in the cohort discontinuing therapy and opting to cryopreserve all embryos, clinical pregnancy was excluded as a main objective.

Notwithstanding the translational importance of the present results, many limitations must be recognised. The observational nature of the research prevents any inferences on a causal link between intrafollicular nitric oxide bioavailability and FSHR polymorphism. Although convergent experimental and clinical literature supports the biologically plausible relationships observed, functional validation within mechanistic models is crucial to definitively demonstrate causality.

The sample size constrains the statistical power for subgroup analysis, especially when stratifying patients by FSHR genotype, but it is comparable to previous molecular studies examining follicular fluid. This restriction highlights the need for larger, multicentre cohorts to confirm the reproducibility of genotype-specific intrafollicular nitric oxide profiles and may clarify the reasons for the lack of statistically significant variations in certain systemic endocrine markers.

Third, rather of assessing nitric oxide signalling in real time, the researchers analysed NO_2_-NO_3_ concentrations, which are stable oxidation products, to evaluate the availability of nitric oxide. This approach inadequately encompasses the spatiotemporal intricacies of nitric oxide dynamics within the follicular milieu, despite its prevalent use and utility in therapeutic contexts. Furthermore, follicular competence may be affected by other redox balance modulators that were not concurrently assessed, such as reactive oxygen species, antioxidant capacity, and eNOS uncoupling status.

The kind and dosage of gonadotropin were established according to usual clinical practice, without genotype-guided stimulation regimens. Thus, it was impractical to explicitly evaluate putative relationships among intrafollicular signalling, gonadotropin formulation, and FSHR genotype. Additional study using pharmacogenomic stratification is essential to ascertain if tailored stimulation approaches may alter nitric oxide bioavailability in a genotype-dependent fashion.

Nonetheless, these constraints outline certain paths for further investigation. Future research must combine FSHR genotyping with extensive intrafollicular profiling, including nitric oxide metabolites, angiogenic factors, and oxidative stress markers, to provide a full map of follicular competence. To clarify the unique interactions of FSHR variations with PI3K/AKT, ERK, and β-arrestin-dependent pathways in modulating eNOS activity and nitric oxide production, simultaneous mechanistic investigations in human granulosa cells and follicle-derived models are necessary.

Future therapeutic studies should assess whether genotype- and microenvironment-targeted ovarian stimulation improves qualitative outcomes, such as oocyte maturity, embryo developmental potential, and cumulative live birth rates, rather than only concentrating on oocyte number. Standardised follicular fluid tests and expedited genotyping platforms may enable the real-time tailoring of stimulation protocols.

The incorporation of intrafollicular functional indicators with genetic background signifies a crucial progression towards precision medicine in assisted reproduction. Future research may transform the FSHR-nitric oxide axis into a therapeutically relevant framework for tailored ovarian stimulation by addressing existing constraints via focused experimental and clinical approaches.

## 5. Conclusions

This research, focussing on nitric oxide bioavailability, presents new evidence that the FSHR Asn680Ser polymorphism is associated with both qualitative intrafollicular signalling dynamics and quantitative ovarian response metrics. Our findings surpass conventional evaluations of controlled ovarian stimulation based on endocrine factors and follicle counts, emphasising the essential influence of the follicular milieu on oocyte competence. The correlation between FSHR genotype and fluctuations in follicular fluid NO_2_-NO_3_ levels, as well as embryological results, substantiates this claim. The current observations support the concept that unique intrafollicular signalling characteristics are defined by FSHR polymorphisms rather than particular alterations in gonadotropin sensitivity. FSHR-mediated signalling may regulate vascular, metabolic, and redox pathways that directly affect oocyte maturation and fertilisation potential, as shown by the associations between the Ser/Ser genotype, nitric oxide dynamics, and enhanced embryological metrics. This methodology elucidates why FSHR genotyping is not consistently effective in assessing crude clinical objectives such as oocyte count or pregnancy rates.

This work enhances the existing data supporting the shift towards precision medicine in assisted reproduction by including genetic variability with functional intrafollicular indicators. The FSHR-nitric oxide axis signifies a potential biological link between gonadotropin signalling and follicular competence, clarifying the processes that contribute to interindividual variability during ovarian stimulation. Our results highlight the need to reevaluate the definition and evaluation of ovarian response in IVF, shifting from quantity-focused classifications to physiologically based oocyte quality criteria. The integration of intrafollicular microenvironmental characterisation with FSHR genotyping may signify a crucial leap in creating tailored stimulation programmes that enhance both effectiveness and reproductive potential.

## Figures and Tables

**Table 1:** Baseline demographic, hormonal, and embryological characteristics of the study population.

Variable	Mean ± SD
Number of patients	89
Age (years)	38.4 ± 6.0
Luteinizing hormone (LH, mIU/mL)	7.15 ± 10.79
Estradiol (E2, pg/mL)	1674.53 ± 1444.64
Follicular fluid NO_2_-NO_3_ (µM)	25.45 ± 23.78
Mean number of oocytes	6.6 ± 5.4
Metaphase II (MII) oocytes	5.3 ± 4.0
Two-pronuclear (2PN) embryos	3.7 ± 3.6

Fundamental embryological, hormonal, and demographic characteristics of the whole research cohort (*n* = 89). Continuous variables are represented as mean ± SD. Nitric oxide metabolites in follicular fluid are measured in micromolar (µM) and expressed as the aggregate of nitrate and nitrite concentrations (NO_2_-NO_3_). LH, E2, MII, and 2PN. Upon reviewing the data, the most significant outliers were eliminated.

**Table 2:** Baseline hormonal parameters according to FSHR Asn680Ser genotype.

Hormone	Carriers (Asn/Asn + Asn/Ser) Mean ± SD	Ser/Ser Mean ± SD	*p*-Value
FSH, (IU/L)	6.99 ± 4.39	8.04 ± 7.51	0.332
E2, (pg/mL)	1982.51 ± 1521.28	2646.38 ± 1980.15	0.145
LH (IU/L)	3.54 ± 5.78	5.69 ± 13.90	0.880
PRG, (ng/mL)	1.14 ± 2.24	2.31 ± 3.89	0.673

Comparison of baseline and trigger-day hormonal characteristics between Ser/Ser homozygous individuals and carriers of the FSHR Asn allele (Asn/Asn and Asn/Ser). Values are presented as the mean ± SD. The Mann–Whitney U test was used for group comparison. No statistically significant difference was seen between the genotype groups (*p* > 0.05).

**Table 3:** Ovarian response and embryological outcomes according to FSHR Asn680Ser genotype.

Outcome	Carriers (Asn/Asn + Asn/Ser) Mean ± SD	Ser/Ser Mean ± SD	*p*-Value
Follicle diameter (mm)	18.92 ± 2.79	18.27 ± 1.44	0.593
Total oocytes retrieved	7.00 ± 4.15	11.07 ± 7.12	0.030
MII oocytes	5.71 ± 3.22	8.67 ± 4.95	0.046
2PN embryos	3.84 ± 2.62	7.00 ± 4.95	0.016

We analysed the ovarian response and embryological outcomes between Ser/Ser homozygous individuals and carriers of the FSHR Asn allele (Asn/Asn and Asn/Ser). Continuous variables are represented as mean ± SD. The Mann–Whitney U test or the independent-samples t-test was used to compare groups as necessary. Statistically significant differences (*p* < 0.05) were identified in total oocyte yield, metaphase II oocytes, and the quantity of two-pronuclear embryos; however, no significant changes in follicular diameter were noted across genotype groups.

**Table 4:** Intrafollicular nitric oxide metabolite concentrations according to FSHR Asn680Ser genotype.

Parameter	Carriers (Asn/Asn + Asn/Ser) Mean ± SD	Ser/Ser Mean ± SD	*p*-Value
NO_2_-NO_3_ (µmol/L)	85.16 ± 76.99	123.57 ± 121.58	0.319

We examined the concentrations of nitric oxide metabolites in follicular fluid from individuals homozygous for the Ser allele and those possessing the FSHR Asn allele (Asn/Asn and Asn/Ser). We included nitrate and NO_2_-NO_3_ to ascertain the availability of nitric oxide, quantified in micromolar (µmol/L). The results are presented as the mean plus or minus the SD. The Mann–Whitney U test was used for group comparison.

## Data Availability

The data presented in this study are available from the corresponding author upon reasonable request.
